# Research on the robustness of the open-world test-time training model

**DOI:** 10.3389/frai.2025.1621025

**Published:** 2025-08-04

**Authors:** Shu Pi, Xin Wang, Jiatian Pi

**Affiliations:** ^1^National Center for Applied Mathematics in Chongqing, Chongqing Normal University, Chongqing, China; ^2^Chongqing Changan Automobile Company Limited, Chongqing, China

**Keywords:** adversarial attacks, testing time poisoning, robustness, open world learning, test-time training/adaptation

## Abstract

**Introduction:**

Generalizing deep learning models to unseen target domains with low latency has motivated research into test-time training/adaptation (TTT/TTA). However, deploying TTT/TTA in open-world environments is challenging due to the difficulty in distinguishing between strong out-of-distribution (OOD) samples and regular weak OOD samples. While emerging Open-World TTT (OWTTT) approaches address this challenge, they introduce a new vulnerability: test-time poisoning attacks. These attacks differ fundamentally from traditional poisoning attacks that occur during model training, as adversaries cannot intervene in the training process itself.

**Methods:**

In response to this threat, we design a novel test-time poisoning attack method specifically targeting OWTTT models. Capitalizing on the fact that model gradients dynamically change during testing, our method employs a single-step query-based approach to dynamically generate and update adversarial perturbations. These perturbations are then input into the OWTTT model during its adaptation phase.

**Results:**

We extensively test our attack method on an OWTTT model. The experimental results demonstrate a significant vulnerability, showing that the OWTTT model's performance can be effectively compromised by our test-time poisoning attack.

**Discussion:**

Our findings reveal that OWTTT algorithms lacking rigorous security assessment against such attacks are unsuitable for real-world deployment. Consequently, we strongly advocate for the integration of defenses against test-time poisoning attacks into the fundamental design of future open-world test-time training methodologies.

## 1 Introduction

The distribution gap between training and testing data poses great challenges to the generalization of modern deep learning methods (Joaquin et al., 2008; Ben-David et al., 2010). To improve the generalization of the model to testing data that may feature a different data distribution from the training data, domain adaptation has been extensively studied (Wang and Deng, 2018) to learn domain-invariant characteristics. However, the existing unsupervised domain adaptation paradigm requires simultaneous access to the data of both the source and the target domain with an offline training stage (Ganin and Lempitsky, 2015; Tang and Jia, 2020). In a realistic scenario, access to target domain data may not become available until the inference stage, and an instant prediction on testing data is required without further ado. Therefore, these requirements lead to the emergence of a new paradigm of adaptation at test time, a.k.a. test-time training/adaptation (TTT/TTA) (Sun et al., 2020; Wang et al., 2021).

The success of TTT has been demonstrated on many synthesized corrupted target domain data (Hendrycks and Dietterich, 2019), manually selected hard samples (Recht et al., 2019) and adversarial samples (Croce et al., 2022). Recently, many major language models have also been using TTA to adjust their models (Hu et al., 2025). However, there are a number of problems with enabling TTT in open-world (OWTTT). One of the problems is that the target domain may contain testing data drawn from a significantly different distribution, e.g., different semantic classes than source domain, or simply random noise (Li et al., 2023). To address this challenge, Li et al. (2023) developed an adaptive strong OOD pruning to improve the effectiveness of the self-training TTT method, while they further proposed a method to dynamically extend the prototype to represent the strong OOD samples to improve the weak/strong OOD data separation.

While this approach has proven successful in ameliorating this problem, it may introduce a new attack surface for the adversary to tamper with the parameters of the target model by fine-tuning them during testing using potentially malicious samples. To explore this possibility, in this work, we propose a method of test-time poisoning attacks (TePAs) against this models. TePAs (Cong et al., 2024) was proposed by Cong et al. i.e., an adversary aims to degrade a TTA model's performance at test time. Compared to TrPAs, TePAs face the following non-trivial challenges: (i) TrPAs require modification access to the target model's training dataset, while TePAs do not poison the training dataset nor control the training process of the target model. (ii) For TrPAs, poisoned samples are mixed with clean training samples where they can be learned in multiple epochs by the model and become more memorable. or trpa, the poisoned samples are mixed with clean training samples so that the model can learn the poisoned samples at multiple epochs and is easier to memorize. However, considering effectiveness and efficiency, the TTA approach usually uses an update of the model based on one calendar element arriving from each test data, hence the different setup for tepa. (iii) In TePAs, poisoned and benign samples are in the same pipeline, and the model is in a state of dynamic adjustment. (iv) Since TePAs are test-time attacks, the adversary must take into account the query budget to maintain the stealthiness of the attack. (v) To avoid the target models “forgetting” the original task, TTA methods usually only update part parameters of the model. However, for TrPAs, the poisoned samples are used to update the whole model parameters.

In summary, these differences make TePAs harder to succeed than TrPAs.

**Our work**. In this paper, our study aims to demonstrate that current OWTTT methods are prone to tepa. Considering their use in safety-critical applications where a deterioration in their efficacy could result in severe consequences, exposing the model modification right to the adversaries is irresponsible, and taking into account TePAs during the design of OWTTT methods becomes crucial.

We propose a Tepa method for the OWTTT model: Single step query attack data poisoning method (SQDP) which uses queries to dynamically generate perturbations and inputs toxic test samples into the model while querying to cause damage to the model. Experiments show that even when mixed with normal test samples in a ratio of 3:2, only a small number of queries are needed, the attack method still has good results and can produce good results on models that have already received a large number of normal test samples.

Meanwhile, we conduct recovery experiments for the models after the attack using normal samples and find that the models of some datasets cannot be recovered, and the phenomenon remains to be further verified. In summary, we make the following contributions.

- We propose a Tepa method: the single-step query attack data poisoning method.
- We conducted experiments using this method, which show that our attack can effectively degrade the performance of the target model with a small number of queries even with a limited number of poisoned samples and after training the model with a large number of normal samples.
- The experiments show that the OWTTT model is difficult to recover effectively after poisoning with normal samples.

## 2 Background

### 2.1 TTT/TTA

Consider that in some cases we would like models already deployed to the target domain to automatically adapt to the new environment without accessing the source domain data. With these considerations in mind, in response to the demand for adaptation to arbitrary unknown target domain with low inference latency, test time training/adaptation (TTT/TTA) (Sun et al., 2020; Wang et al., 2021) have emerged (Li et al., 2023).

We first give an overview of the self-training based TTT paradigm, following the protocol defined in Su et al. (2022). In specific, we define the source and target n datasets as *D*_*s*_ = {_*x*_*i*_, *y*_*i*_}*i* = 1…*N*_*s*__ with label space *C*_*s*_ = {1…_*K*_*s*_}*i* = 1…*N*_*s*__ and *D*_*t*_ = {_*x*_*i*_, *y*_*i*_}*i* = 1…*N*_*t*__ with label space *C*_*t*_ = {1…_*K*_*s*_, *K*_*s*+1_...*K*_*s*+_*K*__*t*__}*i* = 1…*N*_*t*__. In closed-world TTT. *C*_*t*_ = *C*_*s*_, while *C*_*s*_⊆*C*_*t*_ is true under open-world TTT. We further denote the representation learning network as $z_{i}=f\left(x_{i};\theta \right)\in {\mathbb{R}}^{D}$ and the classifier head as *h*(*z*_*i*_; ω, β). Test-time training is achieved by updating the representation network and/or classifier parameters on the target domain dataset *D*_*t*_.

TTT is often realized by three types of paradigms. Self-supervised learning in the testing data enables adaptation to the target domain without considering any semantic information (Sun et al., 2020; Liu et al., 2021). Sun et al. (2020) proposed a method consisting of a main task and a self-supervised auxiliary task. The main task and the auxiliary task share the feature extraction module. The two tasks are trained together during training, and only the auxiliary task updates the model parameters during testing. Liu et al. (2021) addressed the problem that TTT can cause severe overfitting of the updated encoder to the self-supervised learning task in the absence of any constraints on feature distribution and proposed imposing a distribution-based constraint during the test phase training period so that the feature distribution of the test data is close to the feature distribution of the training domain.

Self-training reinforces the prediction of the model in unlabeled data and has been shown to be effective for TTT (Wang et al., 2021; Chen et al., 2022; Liang et al., 2020; Goyal et al., 2022; Lee et al., 2025). Wang et al. (2021) made adjustments to model parameters by minimizing the loss of entropy in model output during the testing phase, while reducing the hardware burden by updating only normalized statistics and affine parameters for all layers and channels. Liang et al. (2020) divided the model into a feature extractor module and a classifier module, and fine-tuned the feature extractor module with the target domain data in the hope of generating source-like representations for the target domain samples.

Lastly, distribution alignment provides another viable approach toward TTT by adjusting model weights to produce features following the same distribution as the source domain (Su et al., 2022; Liu et al., 2021). Su et al. (2022) proposed TTAC by matching the statistics of the target clusters with those of the source clusters and updating the target statistics by using a moving average of the filtered pseudo-labels.

Recent research also exists on methods that do not require gradient descent on the model (Niu et al., 2024; Khurana et al., 2021). Niu et al. (2024) proposed a method that does not require gradient updates to the model. The method targets the transformer-vit model by inserting several embeddings to optimize learning cues during the testing process and improving the derivative-free optimizer covariance matrix adaptation (CMA) evolutionary strategy to achieve the purpose without updating the gradient. Khurana et al. (2021), on the other hand, computed the distribution of a single image by augmenting the data of that image with the data of that image, and used this distribution to design AugBN layer instead of the normal BN layer to achieve distribution alignment for a single image.

Despite efforts to develop more sophisticated TTT methods, the certification of the robustness of TTT is still to be fully investigated.

### 2.2 Poisoning attacks and adversarial attacks

#### 2.2.1 Poisoning attacks

Poisoning attacks are one of the most dangerous threats to ML models (Carlini and Terzis, 2022; Yang et al., 2017). These attacks assume that the adversary can inject poisoned samples into the ML model's training dataset. The assumption is reasonable, as the training datasets of ML models are usually collected from the Internet and it is hard to detect the poisoned samples manually given the size of the dataset. In poisoning attacks, the adversary's goal is to degrade the performance of the model on a validation dataset *D*_*val*_ through some malicious modifications *A* to the training data $D_{train}$ as:


(1) $$\begin{array}{l} \;\underset{A}{\max}L\left(D_{val};\theta^{*}\right)\\ where\;\theta^{*}=arg\underset{\theta }{\min}L\left(A\left(D_{train}\right);\theta \right) \end{array}$$

After being trained on the poisoned dataset $A\left(D_{train}\right)$, the model's performance degrades at test time (Pang et al., 2021).

Poisoning attacks can be broadly grouped into two categories, untargeted poisoning attacks (Muñoz-González et al., 2017; Yang et al., 2017) and targeted poisoning attacks (Biggio et al., 2012; Shafahi et al., 2018). The goal of untargeted poisoning attacks is to reduce the overall performance of the target model. The goal of targeted poisoning attacks is to force the target model to perform abnormally on a specific input class. Backdoor attacks (Pang et al., 2020) are a special case of targeted poisoning attacks in which poisoned target models only misclassify samples that contain specific triggers (Cong et al., 2024). Vasu et al. (2021) proposed an attack method that will not be restricted to model categories, i.e., gradient-based label flipping attack on binary classification models. The proposed attack method is not restricted to model categories, which means that it can be applied to different binary classification models with good portability. For special types of data, Ma et al. also propose effective attacks. To address the problem that pairwise ranking is vulnerable to poisoning attacks, Khurana et al. (2021) proposed a poisoning attack method that can significantly degrade the performance of the sorter, that is, poisoning attack on pairwise comparison estimation. The poisoning attack for pairwise ranking proposed by the authors is a data poisoning attack that can be applied to all attack models with strong robustness. However, all of the above poisoning attack methods are for offline data, and some of them rely on the model's labeling, which is not applicable to test time training/adaptation poisoning.

Recently, Test-Time Poisoning (TePAs) (Cong et al., 2024) was proposed by Cong et al. The attacker aims to degrade the performance of the TTA model at test time. However, there are fewer current studies in this direction, and most of them are untargeted poisoning attacks. This study in this paper focuses on targeted poisoning attacks and for TTT/TTA under OWTTT, which is closer to real-world scenarios.

#### 2.2.2 Adversarial attacks

Adversarial attacks aim to find a perturbed example *x*^*adv*^ around *x* which can be misclassified by the model. Such *x*^*adv*^ is called an adversarial example. Find such adversarial examples can be formulated as the following constrained optimization problem:


(2) $$\begin{array}{l} x^{adv}=arg\underset{x^{\prime }}{\max}L\left(x^{\prime },y;\theta \right)\\ \;s.t.∥x^{\prime }-x{∥}_{p}\leq \epsilon \end{array}$$

where y is the ground-truth label, ∥.∥_*p*_ is the *l*_*p*_-norm, and *L*(.) is the loss.

Adversarial attacks can be roughly divided into four categories: gradient-based, score-based, transfer-based, and decision-based attacks.

Most existing attacks rely on detailed model information including the gradient of the loss w.r.t. the input. Examples are the Fast-Gradient Sign Method (FGSM), the Basic Iterative Method (BIM) (Kurakin et al., 2018), DeepFool (Moosavi-Dezfooli et al., 2016), the Jacobian-based Saliency Map Attack (JSMA) (Papernot et al., 2016), Houdini (Cisse et al., 2017), and the Carlini & Wagner attack (Carlini and Wagner, 2017). Goodfellow et al. (2014) proposed the FGSM method, which works by computing the gradient of the input loss function and generating a small perturbation by multiplying a small selected constant by the sign vector of the gradient. BIM (Kurakin et al., 2018) performs multiple small perturbations in the direction of increasing the gradient in an iterative manner and recalculates the direction of the gradient after each small step. Moosavi-Dezfooli et al. (2016) proposed a new method DeepFool without limiting the range of original sample perturbations, which is an early adversarial sample generation method that can generate perturbations smaller than the fast gradient attack. DeepFool first initializes the original image and assumes that the decision boundaries of the classifier limit the results of the image classification, and then, through each iteration, performs multiple steps of small perturbations along the decision direction of the decision boundary, gradually moving the classification result to the other side of the decision boundary, making the classifier misclassification.

Some attacks are more agnostic and only rely on the predicted scores (e.g., class probabilities or logits) of the model. On a conceptual level, these attacks use the predictions to numerically estimate the gradient. This includes black-box variants of JSMA (Narodytska and Kasiviswanathan, 2016) and of the Carlini & Wagner attack (Chen et al., 2017) as well as generator networks that predict adversaries (Hayes and Danezis, 2017). JSMA (Narodytska and Kasiviswanathan, 2016) proposed Jacobi based significance map attack (JSMA). Instead of utilizing the gradient information of the loss function of the model output, JSMA uses the probabilistic information of the model output categories for backpropagation to obtain the gradient information and then constructs adversarial significance maps for the purpose of the attack. Chen et al. (2017) proposed three adversarial attack methods (*L*_0_ attack, *L*_2_ attack, and *L*_∞_ attack) to find perturbations that minimize various similarity measures.

Transfer-based attacks do not rely on model information, but need information about the training data. This data is used to train a fully observable substitute model from which adversarial perturbations can be synthesized (Nayebi and Ganguli, 2017). They rely on the empirical observation that adversarial examples often transfer between models. If adversarial examples are created on an ensemble of substitute models, the success rate on the attacked model can reach 100% in certain scenarios (Liu et al., 2016).

Decision-based adversarial attacks are based entirely on the final decision of the model (Brendel et al., 2018), which is closer to the black-box model in real-world scenarios, and at the same time, it does not require a lot of knowledge of attack models, which makes it easy to migrate attacks during implementation.

## 3 Methodology

**Figure 1:**
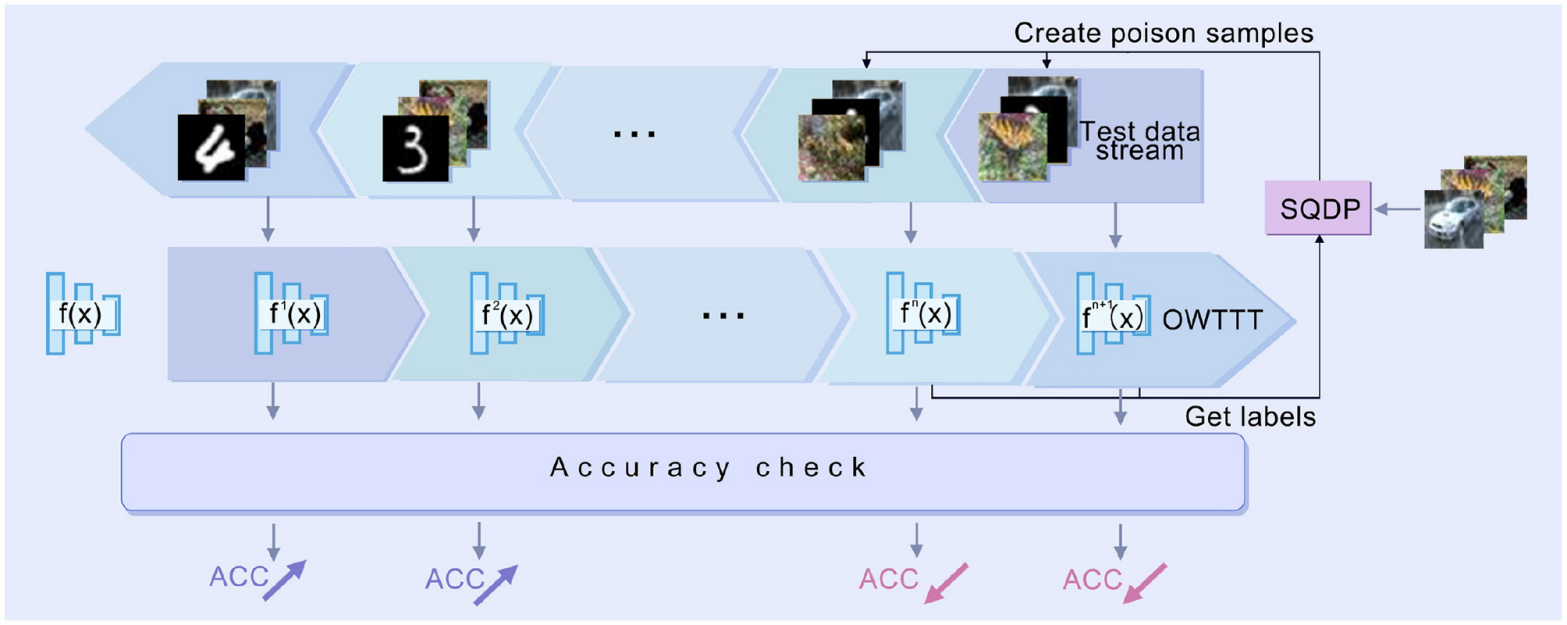
Workflow of SQDP. The adversary uses SQDP to generate poisoned samples which will be fed into the test data stream. The target model f will be updated via OWTTT methods to *f*_*t*_ (the blue one) according to the arrived test data. When meeting benign samples, the performance of *f*_*t*_(*Acc*) will be improved. However, the poisoned samples could degrade the prediction ability of f.

In this chapter, we first review the method of boundary attack. Then we introduce the open-world TTT method based on prototype extension. Finally, we introduce how to apply Single-step Query-attack Data Poisoning(SQDP) to degrade the performance of the model. The overall workflow of SQDP is illustrated in Figure 1.

### 3.1 Open-world TTT algorithm

When calculating strong OOD samples to estimate the target domain distribution, methods based on distribution alignment will be affected. The global distribution alignment (Liu et al., 2021) and the category distribution alignment (Su et al., 2022) can be affected and lead to an incorrect distribution of features.

Therefore, Li et al. proposed an open-world TTT method based on prototype expansion. This method has developed a super parameter-free method to trim strong OOD samples, defining a strong OOD score for each test sample:


(3) $$\begin{array}{l} os_{i}=1-\underset{p_{k}\in P_{s}}{\max}<f\left(x\right),p_{k}> \end{array}$$

The function *f*(*x*) extracts features from the target and *p*_*s*_ represents the cluster centers of various class features in the prototype clustering pool. Then, by using a certain step exhaustive method to minimize the algorithm (Equation 4), we obtain the threshold τ to separate strong OOD and weak OOD data, where $N^{+}\in \overset{n}{\sum }l\left(os_{i}>\tau \right)$, $N^{-}\in \overset{n}{\sum }l\left(os_{i}\leq \tau \right)$:


(4) $$\begin{array}{l} \underset{\tau }{\min}\frac{1}{N^{+}}\underset{i}{\sum }{\left[os_{i}-\frac{1}{N^{+}}sum_{j}l\left(os_{j}>\tau \right)os_{j}\right]}^{2}\\ +\frac{1}{N^{-}}\underset{i}{\sum }{\left[os_{i}-\frac{1}{N^{-}}sum_{j}l\left(os_{j}\leq \tau \right)os_{j}\right]}^{2} \end{array}$$

Simultaneously, dynamically expand the prototype pool to include prototypes representing strong out-of-distribution (OOD) samples. Then, self-training was applied to the source domain prototypes and strong OOD prototypes to create a larger gap in the feature space between the weak and strong OOD samples. The losses of self-training are as algorithm (Equation 5), while *N*(μ_*s*_, Σ_*s*_) is the Gaussian distribution for the source domain feature, *N*(μ_*t*_, Σ_*t*_) is the Gaussian distribution for the target domain feature


(5) $$\begin{array}{l} L_{PC}=-\underset{k\in C_{s}}{\sum }I\left({ŷ}_{i}=k\right)log\frac{exp\left(\frac{<p_{k},z_{i}>}{\theta }\right)}{\sum_{l\in C_{s}}exp\left(\frac{<p_{l},z_{i}>}{\theta }\right)}\\ \;-\underset{k\in C_{t}}{\sum }I\left({ŷ}_{i}=k\right)log\frac{exp\left(\frac{<p_{k},z_{i}>}{\theta }\right)}{\sum_{l\in C_{s}+1}exp\left(\frac{<p_{l},z_{i}>}{\theta }\right)} \end{array}$$

### 3.2 Single-step query-attack data poisoning

Traditional adversarial attacks target models whose gradients are unmetered, and most methods generate adversarial samples from either acquired gradient information or inferred gradient information. However, OWTTT methods continuously update their models based on test data, so the gradients of their models are not constant. Also, because of the existence of strong and weak OOD clustering pools, its gradient information is more difficult to simulate with agent models. Therefore, it is a great challenge to generate samples for models with changing gradient information that can cause a misdiagnosis of the model. Compared to other methods, the query attack can dynamically obtain the boundary information of the model while performing the query, and at the same time requires less model information, so the Single-step Query-attack Data Poisoning (SQDP) method is based on this.

The SQDP is based on boundary attack (Brendel et al., 2018). It is initialized from a point that is already adversarial and then performs a random walk along the boundary between the adversarial and the non-adversarial region such that (1) it stays in the adversarial region and (2) the distance toward the target image is reduced. In other words it perform rejection sampling with a suitable proposal distribution *P* to find progressively smaller adversarial perturbations η_*k*_ according to a given adversarial criterion *c*(:) (Brendel et al., 2018). η_*k*_ is sampled from *N*(0, 1) and then processed to satisfy the following conditions:

- The perturbed sample lies within the input domain,

(6) $$\begin{array}{l} {õ}_{i}^{k-1}+\eta_{i}^{k}\in \left[0,255\right] \end{array}$$

- The perturbation has a relative size of δ,

(7) $$\begin{array}{l} ∥\eta^{k}{∥}_{2}=\delta \cdot d\left(o,{õ}^{k}\right) \end{array}$$

- The perturbation reduces the distance of the perturbed image toward the original input by a relative amount ϵ,

(8) $$\begin{array}{l} d\left(o,{õ}^{k-1}\right)-d\left(o,{õ}^{k-1}+\eta^{k}\right)=\epsilon \cdot d\left(o,{õ}^{k-1}\right) \end{array}$$

In practice, it is difficult to sample from such distributions, so a simpler heuristic is used here: first, we sample from an iid Gaussian distribution η^*k*^*N*(0, 1), and then rescale and clip the samples so that Equations 6, 7 hold. In the second step, we project η^*k*^ onto the sphere around the original image o such that *d*(*o*, õ^*k*−1^)−*d*(*o*, õ^*k*−1^+*eta*^*k*^) = ϵ·*d*(*o*, õ^*k*−1^) and Equation 6 hold. We refer to this as the orthogonal perturbation and use it later in the hyperparameter tuning. In the last step, we make a small shift to the original image so that Equations 6, 8 hold. For high-dimensional inputs and small δ; σ the constraint (Equation 7) will also hold approximately.

Unlike the general query attack, our goal is not to generate adversarial samples, but to degrade the model performance by feeding poisoned samples to the model, while taking into account the dynamics of the model gradient, we fix the number of queries, and at the same time, even if a certain sample is queried for its being a toxic sample in a certain query, it is still queried and adjusted the next time.

Single-Step Query Attack Data Poisoning (SQDP), as an adversarial attack paradigm designed for Open-World Test-Time Training (OWTTT) scenarios, formalizes its execution flow into a three-phase iterative architecture: poisoned sample generation, query mixing with label mapping, and dynamic sample updating. This mechanism adaptively adjusts perturbation strategies through active querying of model feedback, with its core advantage lying in independence from gradient information. This characteristic ensures the robustness of the attack in gradient-dynamic environments induced by test-time training.

- **Poisoned sample generation**. Based on the perturbed sample õ^*k*−1^ from initialization or step *k*−1, generate candidate poisoned samples:

(9) $$\begin{array}{l} {õ}_{i}^{k}={õ}_{i}^{k-1}+\eta_{i}^{k} \end{array}$$

where $\eta_{i}^{k}\sim P\left({õ}^{k-1}\right)$ is random perturbation sampled from proposal distribution and complies with the provisions of Equations 6–8. The method of $\eta_{i}^{k}$ generation is introduced in the third paragraph of this section.

- **Query mixing and label mapping**. To simulate data heterogeneity in open-world environments, a hybrid dataset strategy constructs query inputs:

(10) $$\begin{array}{l} D_{\text{mixed}}=\alpha D_{\text{poison}}+\left(1-\alpha \right)D_{\text{clean}}\;\left(0.0\leq \alpha \leq 1.0\right) \end{array}$$

where α is the preset mixing ratio, and $\alpha D_{\text{poison}}={õ}^{k}$. Then feed candidate poisoned samples to model and obtain prediction:

(11) $$\begin{array}{l} {ỹ}_{\text{mixed}}^{k}=f\left(D_{\text{mixed}}\right) \end{array}$$

This achieves dual objectives:
- Model poisoning attack: Induce the model to output error labels on õ^*k*^ to reduce the performance of the model.
- Mapping $y_{\text{i}}^{k}$ to *c*_*i*_, while *c*_*i*_ refers to the true label of ${õ}_{i}^{k}$:

(12) $$\begin{array}{l} M^{k}=\left\{\left(c_{i},{ỹ}_{i}^{k}\right)|y_{i}^{k}=f\left({õ}_{i}^{k}\right)\right\} \end{array}$$

- **Sample update**. Update perturbed samples based on attack result via Equation 13:

(13) $${\tilde{o}}_{i}^{k}=\{\begin{array}{ll} {\tilde{o}}_{i}^{k-1} & \text{if }{\tilde{y}}_{i}^{k}=c_{i}\;and\;(c_{i},{\tilde{y}}_{i}^{k})\in {ℳ}^{k}\\ {\tilde{o}}_{i}^{k-1}+\eta_{i}^{k} & \text{otherwise} \end{array}$$

The update strategy follows the following principles: when the disturbance successfully leads to misclassification, keep the current disturbance increase, otherwise keep the image the same as the ${õ}_{i}^{k-1}$ . This feedback driven closed-loop optimization significantly improves the attack efficiency.

In conclusion, the core of SQDP methodology resides in alternately executing the aforementioned three-phase process during model testing. Through iterative query-feedback mechanisms, it achieves progressive degradation of the model performance. Compared to conventional gradient-based approaches, its gradient-independent nature effectively overcomes gradient drift caused by test-time training, establishing a novel paradigm for adversarial robustness research in open dynamic environments. Complete algorithmic workflow is detailed in Algorithm 1.

**Algorithm 1:** SQDP.

Require: original image *o* = {_*x*_*i*_}*i* = 1…*N*_, OWTTT model *f*, target image *t*, original labels *c*, dataset *D*
1: function SQDP(*o, c, f, t, D*)
2: while *k* < maximum number of steps **do**
3: draw random perturbation from proposal distribution $\eta_{i}^{k}P\left({õ}^{k-1}\right)$
4: ${ỹ}^{k}=f\left({õ}^{k-1}+\eta_{i}^{k}\right)$
5: $D_{\text{mixed}}=\alpha D_{\text{poison}}+\left(1-\alpha \right)D_{\text{clean}}\;\left(0.0\leq \alpha \leq 1.0\right)\;where\;\alpha D_{\text{poison}}={õ}^{k}$
6: ${ỹ}_{\text{mixed}}^{k}=f\left(D_{\text{mixed}}\right)$
7: $M^{k}=\left\{\left(c_{i},{ỹ}_{i}^{k}\right)|y_{i}^{k}=f\left({õ}_{i}^{k}\right)\right\}$
8: while *i*<*N* **do**
9: if ${ỹ}_{i}^{k}=c_{i}\;and\;\left(c_{i},{ỹ}_{i}^{k}\right)\in M^{k}$ **then**
10: set ${õ}_{i}^{k}={õ}_{i}^{k-1}$
11: else
12: set ${õ}_{i}^{k}={õ}_{i}^{k-1}+\eta_{i}^{k}$
13: end **if**
14: end **while**
15: end **while**
16: end **function**

## 4 Experiments

### 4.1 Settings

#### 4.1.1 Datasets

For the corruption datasets, we selected CIFAR10-C/CIFAR100-C (Hendrycks and Dietterich, 2019) as a small corruption dataset, each containing 10,000 corrupt images with 10/100 categories, and ImageNet-C (Hendrycks and Dietterich, 2019) as a large-scale corruption dataset, which contains 50,000 corruption images within 1,000 categories. We also introduced some style transfer datasets. ImageNet-R (Hendrycks et al., 2021) is a large-scale realistic style transfer dataset that has renditions of 200 ImageNet classes resulting in 30,000 images. Tiny-ImageNet (Pouransari and Ghili, 2014) consists of 200 categories with each category containing 500 training images and 50 validation images. We also introduce some digits datasets. MNIST (LeCun et al., 2002) is a handwritten digit dataset, which contains 60,000 training images and 10,000 testing images. SVHN (Netzer et al., 2011) is a digital dataset in a real street context, including 50,000 training images and 10,000 testing images.

#### 4.1.2 Evaluation metric

**Figure 2:**
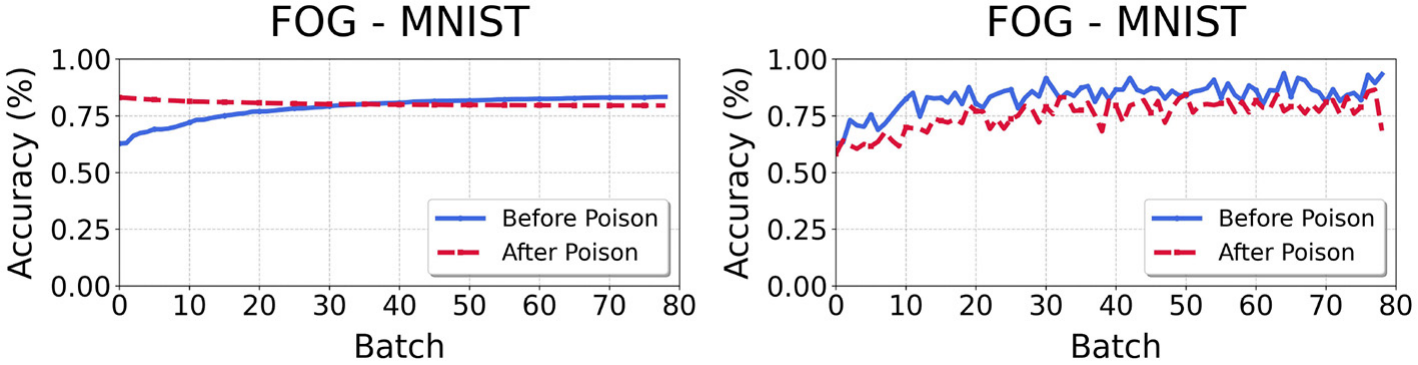
Divergence between cumulative weak OOD accuracy *Acc*_*s* **(left)** and and instantaneous weak OOD accuracy *Acc*_*weak* **(right)**. When $acc_{weak}^{t}$ fails to exceed the decaying *Acc*_*s* threshold, the cumulative metric declines despite actual model improvement.

Our experiments expose a flaw in OWTTT metrics: cumulative indicators (*Acc*_*S*/*N*_) (Li et al., 2023) systematically misrepresent adaptation progress under distribution shift. As Figure 2 demonstrates, when instantaneous weak OOD accuracy fails to exceed the decaying *Acc*_*S*_ threshold ($batch_{weak}^{t}<Acc_{S}^{t-1}$), the legacy metric declines despite rising weak OOD performance—revealing critical temporal metric discordance.

To establish weak OOD generalization as the primary evaluation standard, we adjusted *Acc*_*S*_ propose the core metric *Acc*_*weak*_ as Equation 14, where *B*_*s*_ refers to the weak OOD samples in each batch, ŷ_*i*_ refers to the predicted label and *l*(*y*_*i*_∈*B*_*s*_) is true if *y*_*i*_ is in the set *B*_*s*_:


(14) $$\begin{array}{l} Acc_{weak}=\frac{\sum_{x_{i},y_{i}\in B_{s}}I\left(y_{i}={ŷ}_{i}\right)\cdot I\left(y_{i}\in B_{s}\right)}{\sum_{x_{i},y_{i}\in B_{s}}I\left(y_{i}\in B_{s}\right)} \end{array}$$

Contrasted with the *Acc*_*S*_ as Equation 15, where *C*_*s*_ refers to the cumulative set of all weak OOD samples processed through OWTTT model:


(15) $$\begin{array}{l} Acc_{S}=\frac{\sum_{x_{i},y_{i}\in D_{t}}I\left(y_{i}={ŷ}_{i}\right)\cdot I\left(y_{i}\in C_{s}\right)}{\sum_{x_{i},y_{i}\in D_{t}}I\left(y_{i}\in C_{s}\right)} \end{array}$$

The defining distinction lies in the temporal scope—not data domain. Whereas *Acc*_*S*_ aggregates the accuracy over all historical batches (batches 1 to *t*−1) calculates the instantaneous accuracy exclusively on the current batch.

#### 4.1.3 Training details

Before using SQDP, we pre-train the OWTTT model with the appropriate data and obtain the model's *Acc*_*weak*_ for each training. After that, we use SQDP to poison the model and use normal samples to test the *Acc*_*weak*_ of the model after the poisoning. Below are the parameters of each model:

For the OWTTT part, we follow the parameters specified in Li et al. (2023). We followed the sequential test-time training protocol specified in Su et al. (2022) and choose ResNet-50 (He et al., 2016) as the backbone network for all experiments. For optimization, we choose SGD with momentum to optimize the backbone network. We set the learning rate α = {1*e*−3, 1*e*−4, 2.5*e*−5, 2.5*e*−5, }, the batch size *N*_*B*_ = {256, 256, 128, 128}, λ = {1, 1, 0.4, 0.4}, respectively, for experiments on Cifar10-C, Cifar100-C, ImageNet-C, and ImageNet-R, respectively. To further reduce the effect of incorrect pseudo-labeled, we only use 50% samples with odi far from τ^*^ to perform prototype clustering for each batch. For all experiments, we use temperature scaling δ = 0.1, the length of strong OOD prototypes queue *N*_*q*_ = 100, and the length of moving average *N*_*m*_ = 512.

Although there are known security vulnerabilities in the test time adaptation framework, there is still a lack of research on targeted poisoning attack methods for open world test time training (OWTTT). To establish the baseline evaluation, we used the Diverse Input-FGSM (DIM) attack as a benchmark method, which was used in recent research (Cong et al., 2024). The empirical results show that DIM has a significant destructive effect in a variety of test time training (TTT) and test time adaptation (TTA) paradigms (Cong et al., 2024). For the DIM model, we follow the parameters specified in Cong et al. (2024). We set the perturbation budget ϵ = 32/255 (*l*_∞_-norm) for default. And we set α = 4/255.

For the SQDP model, we used the boundary attack under foolbox,^1^ and we set the parameters as follows: epsilons = 0.3, steps = 100, spherical_step = 0.01, source_step = 0.01, source_step_convergance = 1e-7, step_adaptation = 1.5, and update_stats_every_k = 10. All of the parameters are default except the epsilons and steps.

For the calculation of expenses, we use the A40 graphics card for calculation. For the CIFAR10 and 100 datasets, the OWTTT algorithm consumes 10.21 GB of video memory during runtime, while SQDP attacks the OWTTT model with 13.13 GB of video memory. It takes 82.52 seconds for a hundred queries. For the Imagenet dataset, the OWTTT algorithm consumes 17.24 GB of video memory during runtime, while SQDP attacks the OWTTT model with 40.60 GB of video memory. It takes 84.5 seconds for a hundred queries. Considering the low query time under the current computational load, and the fact that the video memory overhead of this algorithm includes the occupied space of the attacked algorithm, and only the adversarial sample images and target images to be generated need to be loaded during actual operation, the video memory consumption will be greatly reduced. Even graphics cards with lower configurations than A40 can run SQDP algorithm, resulting in lower overall computational overhead.

### 4.2 SQDP against OWTTT models

**Figure 3:**
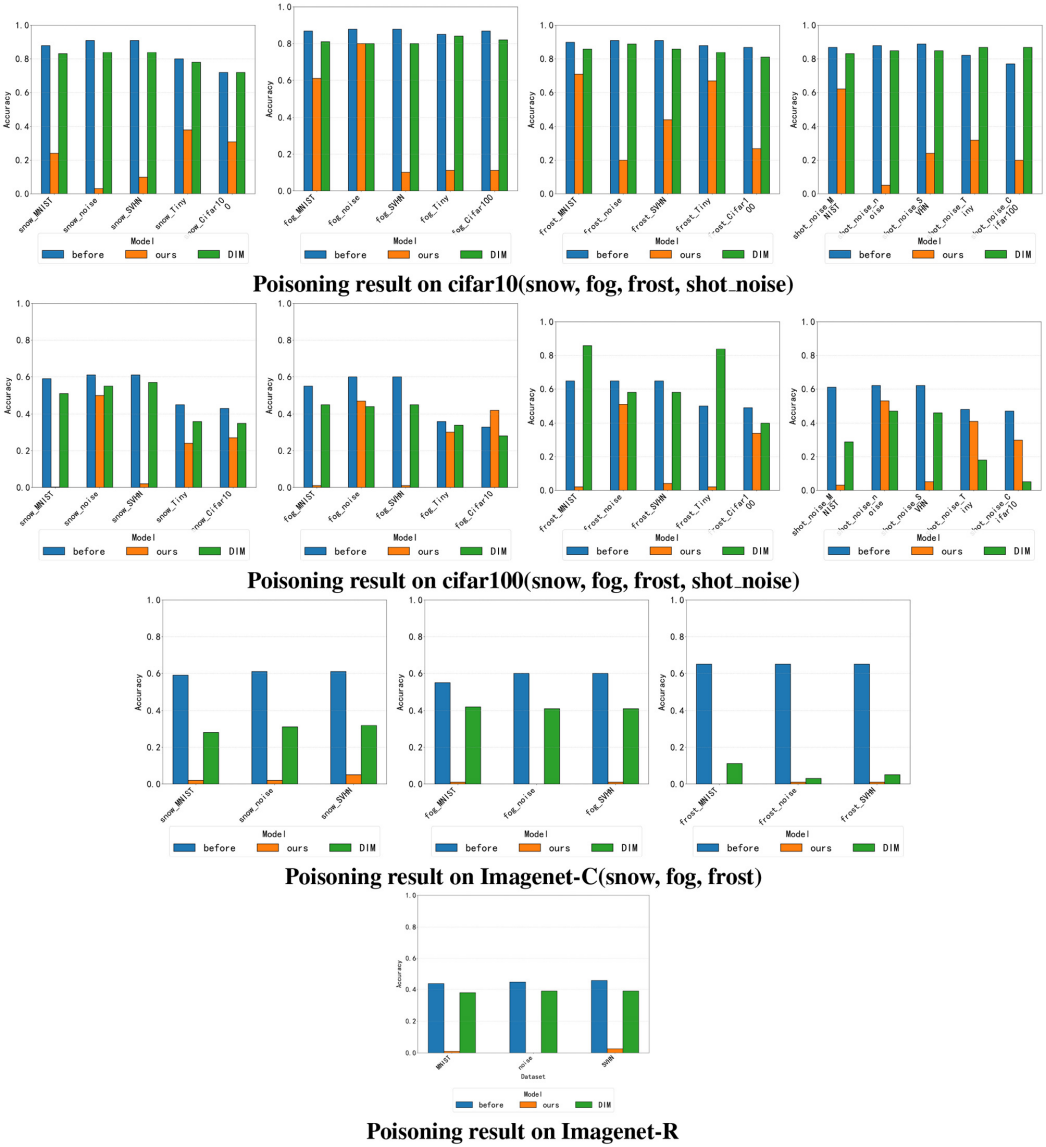
Poisoning results on different datasets. The y-axis and x-axis represent the accuracy (*Acc*_*weak*_) and dataset names of different datasets under SQDP. The x-axis name follows the naming rule of “weak OOD category_strong OOD data.” The above picture effectively proves the feasibility of our method.

We introduce here SQDP against the OWTTT model. In order to fully demonstrate the vulnerability of the OWTTT model to SQDP, we adapt all datasets with poisoned samples and evaluate the impact on the prediction performance. Considering the fluctuation of the results of *Acc*_*weak*_ for a single batch, the comparison of the results takes the average of the last 5 times of the pre-training and the 5 times of the OWTTT model's *Acc*_*weak*_ before testing using normal data after the completion of the SQDP, respectively. The results are shown in Tables 1–4 and Figure 3. The first row in the table represents the category of week ood dataset, and the first column represents the category of strong OOD dataset. There is no weak OOD data in Imagenet-r dataset, so there is no weak OOD data identifier.

**Table 1:** Poisoning results on CIFAR10-C.

	**Snow**			**Fog**			**Frost**			**Shot_noise**		
	**Before**	**Ours**	**DIM**	**Before**	**Ours**	**DIM**	**Before**	**Ours**	**DIM**	**Before**	**Ours**	**DIM**
MNIST	0.88	**0.24**	0.83	0.87	**0.61**	0.81	0.90	**0.71**	0.86	0.87	**0.62**	0.83
noise	0.91	**0.03**	0.84	0.88	**0.80**	0.80	0.91	**0.20**	0.89	0.88	**0.05**	0.85
SVHN	0.91	**0.10**	0.84	0.88	**0.10**	0.80	0.91	**0.44**	0.86	0.89	**0.24**	0.85
Tiny-Imagenet	0.80	**0.38**	0.78	0.85	**0.11**	0.84	0.88	**0.67**	0.84	0.82	**0.32**	0.87
Cifar100	0.72	**0.31**	0.72	0.87	**0.11**	0.82	0.87	**0.27**	0.81	0.77	**0.20**	0.87

Bold value means that the result is better than other comparison models and has achieved better results.

**Table 2:** Poisoning results on CIFAR100-C.

	**Snow**			**Fog**			**Frost**			**Shot_noise**		
	**Before**	**Ours**	**DIM**	**Before**	**Ours**	**DIM**	**Before**	**Ours**	**DIM**	**Before**	**Ours**	**DIM**
MNIST	0.59	**0.002**	0.51	0.55	**0.01**	0.45	0.65	**0.02**	0.86	0.61	**0.03**	0.29
Noise	0.61	**0.50**	0.55	0.60	**0.47**	0.44	0.65	**0.51**	0.58	0.62	0.53	0.47
SVHN	0.61	**0.02**	0.57	0.60	**0.01**	0.45	0.65	**0.04**	0.58	0.62	**0.05**	0.46
Tiny-Imagenet	0.45	**0.24**	0.36	0.36	**0.30**	0.34	0.50	**0.02**	0.84	0.48	0.41	0.18
Cifar10	0.43	**0.27**	0.35	0.33	0.42	0.28	0.49	**0.34**	0.40	0.47	0.30	0.05

Bold value means that the result is better than other comparison models and has achieved better results.

**Table 3:** Poisoning results on Imagenet-C.

	**Snow**			**Fog**			**Frost**		
	**Before**	**Ours**	**DIM**	**Before**	**Ours**	**DIM**	**Before**	**Ours**	**DIM**
MNIST	0.59	**0.02**	0.28	0.55	**0.01**	0.42	0.65	**0.00**	0.11
noise	0.61	**0.02**	0.31	0.60	**0.00**	0.41	0.65	**0.01**	0.03
SVHN	0.61	**0.05**	0.32	0.60	**0.01**	0.41	0.65	**0.01**	0.05

Bold value means that the result is better than other comparison models and has achieved better results.

**Table 4:** Poisoning results on Imagenet-R.

	**Before**	**Ours**	**DIM**
MNIST	0.44	**0.01**	0.38
Noise	0.45	**0.00**	0.39
SVHN	0.46	**0.26**	0.39

Bold value means that the result is better than other comparison models and has achieved better results.

In our study, we first observe that our poisoned samples almost always lead to a significant decrease in the predictive power of the target model, regardless of which combination of strong-OOD and weak-OOD datasets is used. This phenomenon suggests that the quality and characteristics of the data have a non-negligible impact on the performance of the model. By analyzing the experimental results, we find that the poisoned samples can significantly interfere with the normal operation of the model and cause its accuracy to decrease dramatically in the face of unknown data, which also provides an important experimental basis for our subsequent research.

In addition to analyzing the comparability of different combinations of strong-OOD and weak-OOD data, we note that there is a significant difference in the magnitude of model performance degradation. For example, the data in Table 1 shows that when the weak OOD data is SNOW and the strong OOD data is MNIST, the accuracy of the model plummets from 0.88 to 0.24, which shows great vulnerability. Comparatively, when the weak OOD data is replaced with frost, the model performs relatively poorly, with the accuracy similarly dropping to 0.71. This suggests that the model's resistance and adaptability are significantly affected in different data combinations, which depend on the specific characteristics of the dataset.

Finally, our experimental results also show that, compared to the DIM method, our proposed method performs better in most cases. This is evident from our streamlined querying process, where only a small number of queries can effectively degrade the performance of the target model. In our experiments, a significant suppression of the predictive ability of the target model was successfully achieved by performing only 100 queries. This finding not only highlights the effectiveness of our approach but also provides new ideas and approaches for further research and applications.

### 4.3 The recovery of OWTTT model

**Figure 4:**
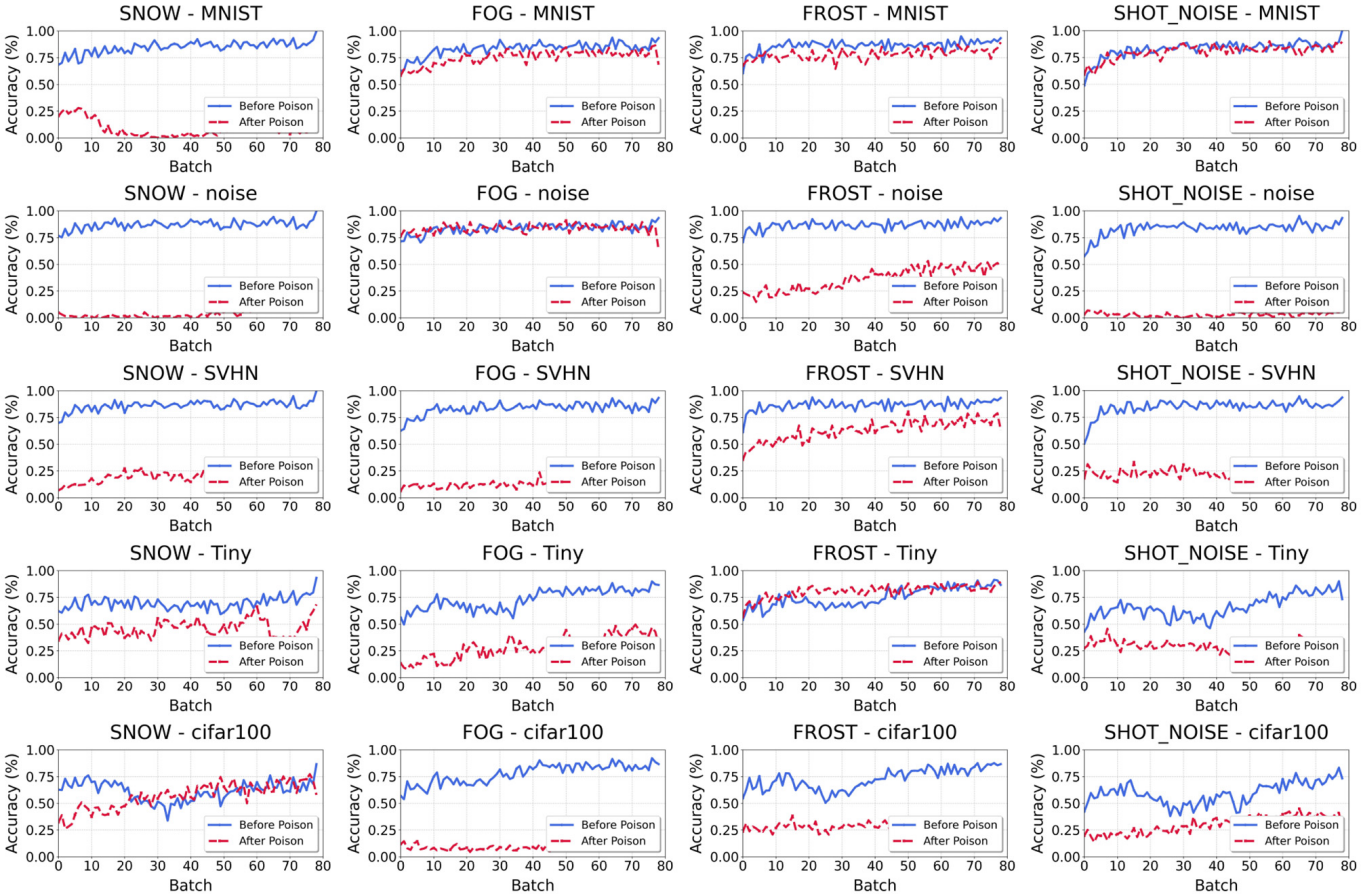
Recover after poisoning. The weak OOD samples are CIFAR-10-C. The y-axis and the x-axis represent the accuracy (*Acc*_*weak*_) of each batch and the number of batch. Each title of the picture follows the naming rule of “weak OOD category_strong OOD data.”

In this study, we investigate the effects of incorporating independent and identically distributed (i.i.d.) samples into the target model post-poisoning. Specifically, we examine a scenario where poisoned samples are introduced first, followed by the feeding of i.i.d. samples. Using the performance on CIFAR-10-C (as illustrated in Figure 4) as a reference, we observe that in some cases, the utility of the model can recover to near normal levels. For example, when combining weak OOD samples from MNIST with strong OOD samples affected by fog, we note that the accuracy of weak OOD samples can return to 0.86, indicating a substantial recovery from the effects of poisoning. This suggests that the degradation in model performance caused by poisoned samples can be substantially mitigated by careful selection of the data fed to the model after the poisoning process.

However, it is important to note that, in most combinations of strong and weak OOD datasets, the efficiency of the model does not exhibit significant recovery. For instance, when the weak OOD sample is MNIST and the strong OOD sample is snow, the accuracy after recovery only reaches 0.05, which is drastically lower than the pre-poisoning performance levels. This result underscores a troubling aspect of model vulnerability; it illustrates that certain combinations of datasets may lead to conditions from which the model cannot effectively recover. Thus, these findings suggest the potential for enduring detrimental effects on the model's predictive capabilities following an attack, raising concerns about the resilience of machine learning models in similar threat scenarios.

Finally, our experimental results show that our proposed method outperforms the DIM method in most cases. In TePAs, the TTA method attacked using the DIM method is recoverable after accepting normal samples; however, the present method makes recovery impossible on some datasets. Note that we effectively degrade the performance of the target model using only a small number of queries. In our experiments, the predictive power of the target model was significantly suppressed with only 100 queries, which proves the effectiveness of our method. At the same time, the unrecoverable nature of the attack shows that the attack method is fatal to the model and needs to be highly emphasized.

### 4.4 Factors that may affect the effectiveness of the attack

In this chapter, we systematically explore the various factors that may affect the effectiveness of an attack. To achieve this, we design a series of experiments utilizing two different datasets: the MNIST dataset as out-of-distribution (OOD) data and the CIFAR-10-C dataset as in-distribution data. Without additional instructions, the rest of the parameters in the experiment are the same as in Section 4.1. Through the experiments in this chapter, we aim to demonstrate the effectiveness of the attack methodology and gain insight into how different factors can change the dynamics of the attack's effectiveness.

#### 4.4.1 The settings of target

The previous attacks used strong OOD data as the target, and added perturbations to the weak OOD data and input them into the model. To confirm whether the target setting has any effect on the attack effect, in this chapter, we set weak OOD as the target, add perturbation to the strong OOD data and input it into the model, and other experimental conditions remain unchanged. The results are shown in Table 5.

**Table 5:** Poisoning results on strong oods.

**Target**	**Snow**	**Fog**	**Frost**	**Shot_noise**
Strong	0.47	0.01	0.04	0.08
Weak	0.24	0.61	0.71	0.62

From the table, it can be seen that there is a significant difference in the attack effect for different datasets with different target settings. Specifically, when the weak OOD data category is set to “snow,” the attack effect partially decreases; while in the other three categories, the attack effect increases significantly. These results suggest that the effect of target setting on attack effectiveness cannot be ignored. However, it is worth noting that even if the target setting is changed, the attack method itself remains valid and does not lead to a fundamental failure of the attack effect. Therefore, differences in target settings do not impede the effectiveness of the attack methods.

#### 4.4.2 Poisoning models without pre-training

In this section, we conduct systematic attack experiments on models that are not pre-trained and provide data on normal samples after the attack to test whether the performance of the model is significantly affected by its performance in the pre-trained state. The experimental results are detailed in Table 6, where the row named origin represents the mean value of acc_weak for the untrained model on the initial 5 normal sample batch sets. The row named after indicates the mean value of acc_weak on the initial 5 normal sample batch for the model after accepting the poisoned samples.

**Table 6:** Poisoning results on models without pre-training.

**Target**	**Snow**	**Fog**	**Frost**	**Shot_noise**
Origin	0.73	0.68	0.73	0.61
After	0.05	0.02	0.00	0.23

**Figure 5:**
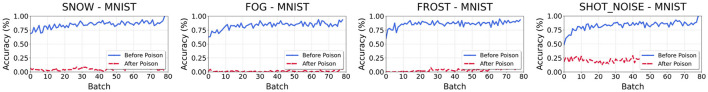
Recover after Poisoning in no per-trained model. The weak OOD samples are CIFAR-10-C. The y-axis and the x-axis represent the accuracy(*Acc*_*weak*_) of each batch and the number of batch. Each title of the picture follows the naming rule of “weak OOD category_strong OOD data.”

The experimental results shown in Figure 5 show that generating poisoned samples against an uninitialized model can effectively reduce its initial accuracy in open-world scenarios, and that this attack does not negatively affect the attack performance of the model. In addition, the attacked model has more difficulty in recovering its performance when faced with normal samples, which further emphasizes the importance of pre-training for model stability and recovery.

#### 4.4.3 The times of queries

**Figure 6:**
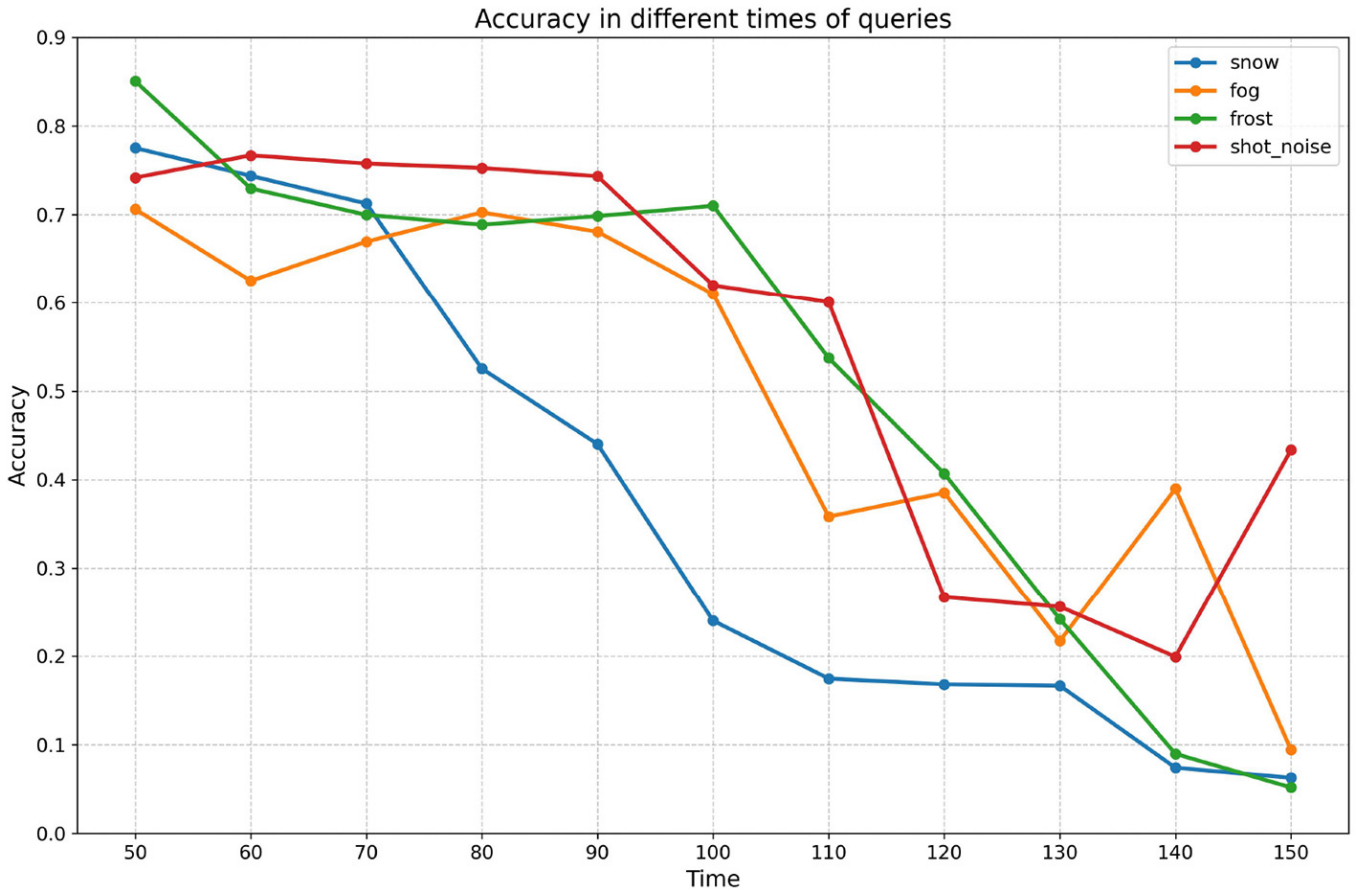
Poisoning results on different queries The y-axis and x-axis represent the accuracy (*Acc*_*weak*_) and query count for different query counts.

In this chapter, we aim to investigate the relationship between attack effectiveness and the number of queries. It is evident that attack effectiveness is closely related to the number of queries; however, the precise nature of this relationship remains to be explored further. To systematically analyze the impact of varying query counts on attack effectiveness, we have designed experiments with the number of queries set at 50, 75, 100, 125, and 150. The experimental results are presented in Table 7 and Figure 6, which will provide significant empirical support for understanding how query counts influence attack effectiveness.

**Table 7:** Poisoning resultes on different queries.

**Times**	**0**	**50**	**60**	**70**	**80**	**90**	**100**	**110**	**120**	**130**	**140**	**150**
Snow	0.88	0.78	0.74	0.71	0.52	0.44	0.24	0.17	0.17	0.17	0.07	0.06
Fog	0.87	0.71	0.63	0.67	0.70	0.68	0.61	0.36	0.39	0.22	0.39	0.09
Frost	0.90	0.85	0.73	0.70	0.69	0.70	0.71	0.54	0.41	0.24	0.09	0.05
Shot_noise	0.87	0.74	0.77	0.76	0.75	0.74	0.62	0.60	0.27	0.26	0.20	0.43

Firstly, the effectiveness of the attacks increases steadily with the number of attacks. Based on the data presented in the table, it is evident that all combinations demonstrate a general decline in accuracy as the number of queries increases. This observation not only indicates the effectiveness of the attacks but also confirms that the decrease in model accuracy during the experiments is not limited to a specific query point; rather, it represents a widespread and systematic phenomenon. This finding suggests that the model consistently exhibits vulnerability in the face of increasing attack frequency. Therefore, we can conclude that the efficacy of the attacks is robust, and the decline in model performance is not an isolated incident, but rather a clear reflection of the cumulative impact of the attacks.

Second, the relationship between the effectiveness of the attack and the number of queries does not grow linearly. In the experiments with snow as the weak OOD dataset, the model accuracy decreases significantly after the 100th query is performed, while the decrease is limited in the first 100 queries, showing that there is a specific query threshold; when the threshold is reached, the attack effectiveness increases significantly. The query threshold also varies across datasets; for example, for the frost dataset, the threshold is not reached until the 125th query. Considering that the frequency of calls against the same interface is usually limited in open-world scenarios, too high a number of queries does not meet the practical application requirements. Therefore, the attack strategy of limiting the number of queries is more applicable in real-world applications and provides a more realistic reference for model security evaluation.

#### 4.4.4 The percentage of mixed samples

In this section, we explore the effect of sample mixing ratio on the proposed method. The data in Table 8 show that there is a significant correlation between the attack effect and the mixing ratio, but the exact pattern of the relationship still needs to be studied in depth. To this end, we will input the generated toxic data into the model according to five different ratios, namely 0.2, 0.4, 0.6, 0.8 and 1.0, with the aim of observing the changes in model performance.

**Table 8:** Poisoning results on different mixed percentage.

**Per**	**0**	**0.2**	**0.4**	**0.6**	**0.8**	**1.0**
Snow	0.88	0.74	0.63	0.24	0.32	0.11
Fog	0.87	0.71	0.68	0.61	0.30	0.09
Frost	0.90	0.74	0.78	0.71	0.46	0.18
Shot_noise	0.87	0.40	0.72	0.62	0.75	0.11

First, we note that the poisoned samples generated at different mixing ratios all have a significant negative impact on the performance of the model. This phenomenon not only clearly demonstrates the effectiveness of the attack method, but also shows that the poisoned samples generated by the method are capable of causing substantial damage to the model even at very low mixing ratios. This important finding highlights the importance of giving high priority to this attack method when performing security evaluations of models, as the magnitude of the potential threat may be much higher than we expect.

Second, we observe that changes in the mixing proportions of different samples directly affect the performance of the attack model. Under most datasets, the model performance generally shows a decreasing trend as the proportion of poisoned samples gradually increases, especially when the weak OOD dataset is snow, fog, and frost. However, when the weak OOD dataset used is shot_noise, the model performance is relatively superior, and only at mixing ratios of 0.2 and 1.0, the model performance can still be maintained at a relatively good level. This result suggests that how to specifically define and select the mixing ratio of the samples is still an important topic worthy of in-depth research, especially in the process of optimizing the model's ability to resist attacks. Further exploration in this research direction will provide a theoretical basis and practical guidance to improve the robustness and security of the model.

## 5 Discussion

The main findings of the study reveal that the robustness of current TTT/TTA models, especially TTT/TTA (OWTTT) models in open-world environments, is in dire need of enhancement and has significant security concerns and risks. Specifically, we propose a Single Query Data Poisoning (SQDP) attack methodology, by which we are able to significantly reduce the accuracy of models on different datasets with only 100 queries. This finding implies the vulnerability of the model against potential attacks. It is worth noting that previous studies (e.g., Tepas) have focused on traditional TTT/TTA models, which are not as effective against attacks in open-world environments. In addition, we observe that some instances of the models that have been attacked by SQDP appear to be unrecoverable by normal samples, which further emphasizes the vulnerability of the models. Due to the fact that the ImageNet dataset contains 1,000 fine-grained object categories (Russakovsky et al., 2015), covering most visual concepts in the real world (Deng et al., 2009), the robustness results validated on this dataset have broad representativeness and transferability (Hendrycks and Dietterich, 2019).

The importance of this finding is not only on the technical level, but also relates to the practical application of TTT/TTA technology in critical areas such as medical diagnosis and autonomous driving. In the current context of rapid development, models with high accuracy and strong adaptability will provide more efficient and reliable solutions in these fields. However, the popularization of technology is accompanied by security risks that cannot be ignored. For example, attacks on models through specific means can lead to significant degradation of model performance, which can have serious consequences. Despite the growing interest in this area, research in this area still appears to be relatively scarce, making the results of this study of great academic and practical significance.

Despite the results of this study, we must also recognize its limitations. First, although the experiments prove the effectiveness of the SQDP attack method, in some cases, when the percentage of poisoned samples is very low, the model performance decreases relatively slowly or requires more queries, increasing the cost of the attack. In addition, this paper does not provide an in-depth discussion of strategies for defending against this attack method, whereas SQDP attacks are more necessary to cope with potentially changing attack methods than traditional adversarial defense strategies.

Based on the findings of this study, future research directions can focus on the following two areas:

- Designing more efficient attack algorithms to generate poisoned samples and execute attacks against the model.
- Exploring practical and effective defense strategies aimed at countering attacks against OWTTT models.

In conclusion, this study clearly demonstrates the possible robustness issues and security risks of OWTTT techniques. We call on researchers to pay more attention to the security issues of AI while pursuing technological advances in order to realize the sustainable development of AI technology.

## 6 Conclusion

In this article, we conducted an in-depth study of targeting test-time poisoning attacks (TePAs) for the Open-world Test-time Training (OWTTT). Specifically, we propose a toxic sample generation framework that relies on query-based adversarial attack techniques to construct disruptive adversarial samples. These adversarial samples are then used as poisoned samples designed to significantly degrade the performance of OWTTT models by maliciously manipulating the inputs to the target model. Through empirical evaluation, our experimental results show that this attack methodology is largely successful in weakening the performance of the target OWTTT model, demonstrating the effectiveness and relevance of our attack strategy.

In addition, we note that the target model has an extremely low probability of recovering its performance after experiencing our attack. This finding reveals the fatal flaws of the OWTTT model in the face of the target test-time poisoning attack, and suggests that the existing models have serious shortcomings in terms of security and robustness. Therefore, how to conduct an effective defense against such attacks becomes an interesting research direction that deserves in-depth exploration. We believe that the research on defense mechanisms for OWTTT models not only has important academic value, but also has practical significance for security enhancement in practical applications.

In conclusion, our study shows that current OWTTT methods are vulnerable to test-time poisoning attacks, a finding that provides important insights for future research. Based on this, we advocate the active integration of defenses against test-time poisoning attacks in the design of future OWTTT methods to enhance the security and robustness of the model. Through such efforts, we hope to promote the further development of the OWTTT field in resisting adversarial attacks and lay the foundation for building more secure and reliable target tracking systems.

## Data Availability

The original contributions presented in the study are included in the article/supplementary material, further inquiries can be directed to the corresponding author.

## Footnotes

^1^ https://github.com/bethgelab/foolbox.git
