# Correction: Research on the robustness of the open-world test-time training model

**DOI:** 10.3389/frai.2025.1682908

**Published:** 2025-08-29

**Authors:** Shu Pi, Xin Wang, Jiatian Pi

**Affiliations:** ^1^National Center for Applied Mathematics in Chongqing, Chongqing Normal University, Chongqing, China; ^2^Chongqing Changan Automobile Company Limited, Chongqing, China

**Keywords:** adversarial attacks, testing time poisoning, robustness, open world learning, test-time training/adaptation

Affiliation: Chongqing Changan Automobile Company Limited, Chongqing, China was omitted for author Xin Wang. This affiliation has now been added for author Xin Wang.

The original version of this article has been updated.

